# Does closed-loop automated oxygen control reduce the duration of supplementary oxygen treatment and the amount of time spent in hyperoxia? A randomised controlled trial in ventilated infants born at or near term

**DOI:** 10.1186/s13063-023-07415-9

**Published:** 2023-06-15

**Authors:** Ourania Kaltsogianni, Theodore Dassios, Allan Jenkinson, Anne Greenough

**Affiliations:** 1 Department of Women and Children’s Health, School of Life Course Sciences, Faculty of Life Sciences and Medicine, King’s College London, London, UK; 2 Neonatal Intensive Care Centre, King’s College Hospital NHS Foundation Trust, London, UK

**Keywords:** Closed-loop automated oxygen control, Term infants, Mechanical ventilation, Hyperoxaemia

## Abstract

**Background:**

Ventilated infants frequently require supplemental oxygen, but its use should be monitored carefully due to associated complications. The achievement of oxygen saturation (SpO_2_) targets can be challenging as neonates experience frequent fluctuations of their oxygen levels that further increase the risk of complications.

Closed-loop automated oxygen control systems (CLAC) improve achievement of oxygen saturation targets, reduce hyperoxaemic episodes and facilitate weaning of the inspired oxygen concentration in ventilated infants born at or near term. This study investigates whether CLAC compared with manual oxygen control reduces the time spent in hyperoxia and the overall duration of supplemental oxygen treatment in ventilated infants born at or above 34 weeks gestation.

**Methods:**

This randomised controlled trial performed at a single tertiary neonatal unit is recruiting 40 infants born at or above 34 weeks of gestation and within 24 h of initiation of mechanical ventilation. Infants are randomised to CLAC or manual oxygen control from recruitment till successful extubation. The primary outcome is the percentage of time spent in hyperoxia (SpO_2_ > 96%). The secondary outcomes are the overall duration of supplementary oxygen treatment, the percentage of time spent with an oxygen requirement above thirty per cent, the number of days on mechanical ventilation and the length of neonatal unit stay. The study is performed following informed parental consent and was approved by the West Midlands-Edgbaston Research Ethics Committee (Protocol version 1.2, 10/11/2022).

**Discussion:**

This trial will investigate the effect of CLAC on the overall duration of oxygen therapy and the time spent in hyperoxia. These are important clinical outcomes as hyperoxic injury is related to oxidative stress that can adversely affect multiple organ systems.

**Trial registration:**

ClinicalTrials.Gov NCT05657795. Registered on 12/12/2022.

**Supplementary Information:**

The online version contains supplementary material available at 10.1186/s13063-023-07415-9.

## Background

Term-born infants require respiratory support for a variety of reasons with an overall incidence of mechanical ventilation of 3.6 per 1000 live births [1]. Mechanical ventilation, although life-saving, is associated with complications including chronic lung disease, abnormal neurodevelopment and increased mortality [2, 3]. Late preterm infants (those born between 34 and 36^+6^ weeks gestation) account for the majority of preterm births and, due to the interruption of normal lung development, are more likely to need mechanical ventilation and at higher risk of all forms of respiratory morbidity when compared to term born neonates [4].

Ventilated neonates frequently require supplemental oxygen, but its use must be monitored carefully due to associated complications. Therefore, oxygen saturation levels (SpO_2_) are continuously monitored in clinical practice and used to guide adjustments to the inspired oxygen concentration (FiO_2_), which are traditionally made manually by neonatal practitioners.

Maintaining peripheral oxygen saturation levels within targets can be challenging as neonates experience frequent fluctuations and episodes of intermittent hypoxaemia or hyperoxaemia [5]. Consequently, clinical staff face a considerable burden of work continuously monitoring and responding to changes in SpO_2_ levels due to the frequency of adjustments needed. Compliance with SpO_2_ targets in oxygen saturation ranges has been variable even within the same patient over time, as well as between patients and centres [6].

Closed-loop automated oxygen control (CLAC) systems work through an algorithm comparing the patient’s SpO_2_ readings with the desired setpoint and calculate an updated value for the inspired oxygen concentration (FiO_2_) that is mechanically adjusted without any human intervention. There are several commercially available devices using different algorithms. An example is the Oxygenie software (SLE) that has been shown to be effective in improving compliance with target achievement in preterm infants and preventing hypoxaemia and hyperoxaemia [7]. In a randomised crossover study, using that device we demonstrated that preterm ventilated infants experienced fewer prolonged desaturations during the closed-loop automated oxygen control period and spent an increased percentage of time within their target SpO_2_ range with fewer manual adjustments to the inspired oxygen concentration [8]. In agreement with the above, a meta-analysis including 13 randomised controlled trials [9], concluded that automated oxygen control systems significantly reduced the percentage of time infants spent in hyperoxia (SpO_2_ > 98%) with fewer manual adjustments to the inspired oxygen concentration. A literature review concluded that the inspired oxygen concentration was reduced more rapidly during automated oxygen control when compared to manual oxygen control [10].

Most of the studies assessing closed-loop automated oxygen control included very preterm or low birth weight infants. Yet, more mature infants are also vulnerable to the risks related to oxygen treatment. In a randomised crossover study in ventilated infants born at or above 34 weeks gestation, we demonstrated that CLAC increased the percentage of time spent within the target range (SpO_2_: 92–96%) (*p* = 0.001), reduced the time spent in hyperoxia (*p* = 0.006) and the duration of hyperoxaemic episodes (*p* = 0.001), with fewer manual adjustments to the inspired oxygen concentration (*p* = 0.004) [11]. In addition, the median FiO_2_ requirement was lower during the automated oxygen control period (*p* = 0.014). It is, therefore, possible and our hypothesis that the use of CLAC will reduce the overall duration of oxygen treatment by reducing the time spent in hyperoxia and facilitating weaning of the inspired oxygen concentration.

The aim of this study is to explore if in ventilated infants born at or above 34 weeks of gestation, the use of closed-loop automated oxygen control compared to standard care reduces the overall duration of supplemental oxygen therapy and the time spent in hyperoxia. These are important outcomes related to respiratory morbidity and the complications arising from oxygen treatment. Our primary outcome is the percentage of time spent in hyperoxia (SpO_2_ > 96%). The secondary outcomes of this study are the duration of supplementary oxygen treatment, the percentage of time with an oxygen requirement exceeding thirty per cent, the number of days on mechanical ventilation and the length of neonatal unit stay.

## Methods

This is a non-blinded, superior, randomised controlled trial. Infants are allocated to parallel groups in a 1:1 ratio.

### Setting

A single tertiary neonatal unit at King’s College Hospital NHS Foundation Trust, London, UK. The unit has previous experience on the use of closed-loop automated oxygen control in the context of trials.

### Inclusion criteria

Inclusion criteria are as follows:

1. Infants delivered at or above 34 weeks of gestational age requiring mechanical ventilation.
2. Recruitment within 24 h of initiation of mechanical ventilation.

### Exclusion criteria

1. Infants delivered before 34 weeks of gestational age.
2. Infants with congenital cyanotic heart disease.
3. Infants on high-frequency oscillatory ventilation (HFOV).
4. Infants who require oxygen saturation targets > 96%, e.g. infants with pulmonary hypertension or pneumothorax.

### Recruitment

Parents or legal guardians of all eligible infants are initially approached by the clinical team taking care of the infant and if they agree, by a researcher. The parents are provided with an information sheet about the study. The researchers answer questions and respond to any concerns in a face-to-face meeting and obtain written informed consent. The study was approved by the Health Research Authority and by the West Midlands-Edgbaston NHS Research Ethics Committee.

### Randomisation

The randomisation sequence is generated by using an online randomisation generator and concealed in sealed opaque envelopes. This is done by a person independent of the research team who is not involved in the study. A member of the research team will enrol participants to their allocation.

### Enrolment

Infants will be enrolled in the study by the research team within 24 h of initiation of mechanical ventilation following parental consent. Infants are randomised to one of the study arms by opening the next envelope.

### Blinding

The study is not blinded. Decisions regarding ongoing care, for example, ventilator settings and interventions such as blood gases and chest radiographs, are made by the clinical team as per the neonatal unit’s guidelines.

### Intervention

At enrolment, participants are randomised to receiving either closed-loop automated oxygen control (intervention group) or manual control of the inspired oxygen concentration. All infants are ventilated using the SLE6000 ventilators and ventilation settings are manually adjusted by the clinical team as per the unit’s protocol. In addition to standard care, infants in the intervention group are connected to the Oxygenie Auto-O_2_ software (SLE). This software uses oxygen saturation levels from the SpO_2_ probe attached to the neonate, which are fed into an algorithm, to automatically adjust the percentage of inspired oxygen to maintain oxygen saturation within the target range. Oxygen saturation readings will be pre- or post-ductal, unless there is a difference between them, and then pre-ductal readings will be used. Manual adjustments including the percentage of inspired oxygen are allowed at any point during the study, if deemed appropriate by the clinical team. No concomitant treatments will be prohibited during the trial.

The nurse-to-patient ratio is according to the unit protocol that is determined on the patient’s acuity.

**Fig. 1:**
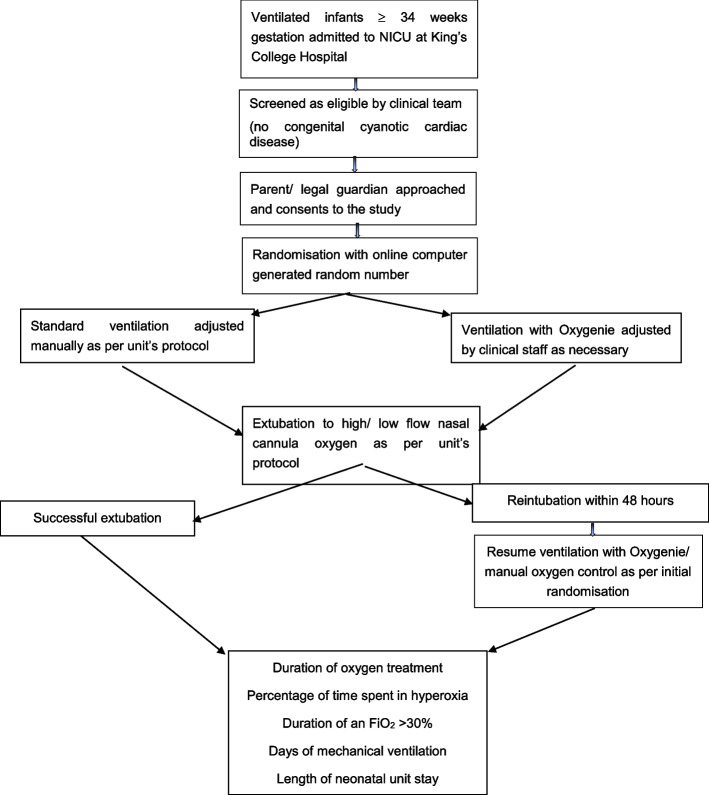
Trial flowchart (Protocol v1.2, 10/11/2022)

Infants are studied from enrolment until successful extubation [12]. Infants who fail extubation and require reintubation within 48 h continue in their initial study arm. Therefore, for infants randomised to the intervention group closed-loop oxygen delivery resumes. If an infant is successfully extubated the study will be completed [11] (Fig. 1).

### Primary outcome measure

The primary outcome measure is the percentage of time spent in hyperoxia (SpO_2_ > 96%).

### Secondary outcomes

The secondary outcomes are the percentage of time spent with an oxygen requirement exceeding 30%, the duration of supplementary oxygen treatment, the number of days on mechanical ventilation and the length of neonatal unit stay.

**Fig. 2:**
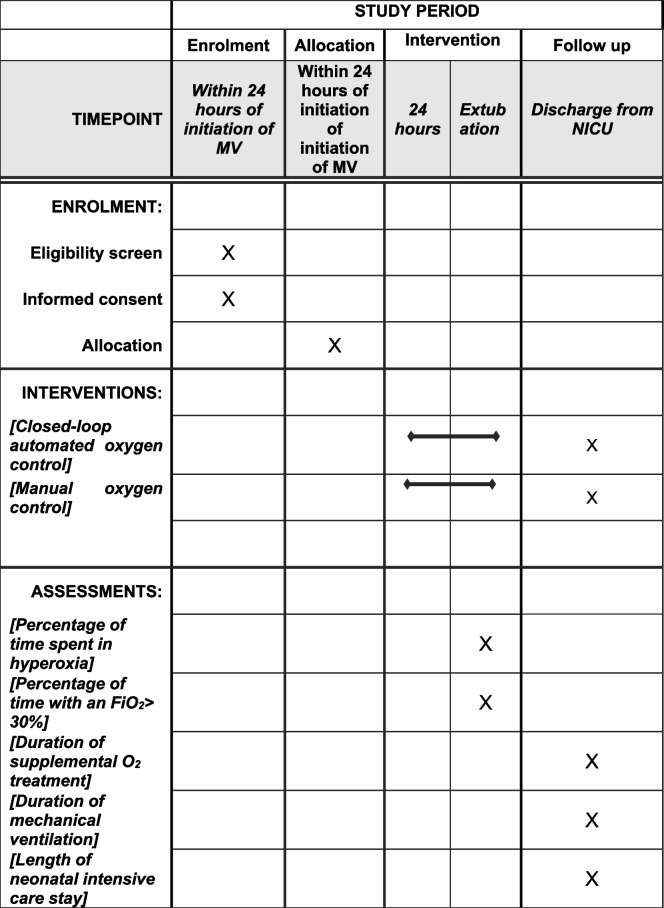
SPIRIT figure of trial interventions and timings

The order of study events is detailed in the Standard Protocol Items: Recommendations for Interventional Trials (SPIRIT) figure in Fig. 2. A SPIRIT checklist is also provided as an Additional file and the protocol is based on the SPIRIT reporting guidelines [13].

### Sample size

In an interim analysis of data from a randomised crossover study on CLAC in ventilated infants born at or above 34 weeks of gestation that was undertaken in our unit, the use of a closed-loop oxygen system was associated with a reduction in the mean FiO_2_ delivery during hyperoxia from 29.6% (manual oxygen control period) to 24.7% (automated oxygen control period, *p* = 0.018), that is a difference of 4.9%. The standard deviation for the FiO_2_ requirement of the infants at the beginning of the automated oxygen control period was 4.7% [14]. Randomisation of 40 infants allowed to detect a difference in the mean FiO_2_ delivery during hyperoxia of 4.9% with a 90% power at the 5% significance level [15].

At the request of the Ethics committee, an interim analysis will be carried out after half the sample have been recruited to review if enough data have been gained at that point. The analysis will be performed by an independent researcher not involved in the project to avoid introducing bias to our data. The research team will be blinded to the results of the interim analysis unless there are any significant findings.

### Data collection

Demographic and outcome data will be collected from the clinical records and recorded under a unique study identifier number. Basic epidemiologic parameters such as gestational age at birth, birth weight, corrected gestational age, postnatal age and weight at study enrolment, use of antenatal steroids, mode of delivery, doses of surfactant administered, birth plurality and diagnoses at enrolment will be recorded for each infant. Oxygen saturation and FiO_2_ data will be downloaded from the ventilators. Paper data will be stored in a locked filing cabinet. Patient de-identified electronic data will be stored in password-protected computers and on encrypted devices.

### Statistical analysis

Data will be compared between infants in the intervention group (closed-loop automated oxygen control) and infants in the control group (manual oxygen control). The data will be assessed for normality; Student’s *t* test will be used for normally distributed data and Mann–Whitney *U* test for skewed data. Categorical data will be assessed using the Fisher two-tailed exact test. The analysis will be undertaken using SPSS software (SPSS Inc, Chicago, IL, USA). Patients’ data will be analysed according to their randomised assignment group. Imputation of missing data will not be performed.

Ventilation settings and oxygen requirements at the beginning of the study will be compared between infants in the intervention group (closed-loop automated oxygen control) and infants in the control group (manual oxygen control). If there are any statistically significant differences indicating different severities of respiratory disease, we will perform subgroup analysis and comparisons between infants with more and less severe lung disease.

### Safety

All participants will receive standard care and monitoring by clinical staff. Ventilator settings will be adjusted and additional investigations such as blood gases and chest radiographs will be performed at the discretion of the clinicians. Therefore, there should not be any risk imposed to the intervention group with the addition of the closed-loop automated oxygen delivery system. The system does not mask large increased oxygen requirements between manual observation recordings, as it activates an alarm when there is an increase in the oxygen requirement ≥ 30% from the basal level alerting the clinical team. All serious adverse events will be recorded on a serious adverse event (SAE) form and will be emailed to the sponsor within one working day of the Chief Investigator (CI) becoming aware of the event. When the event is unexpected and thought to be related to the use of the device, this will be reported by the CI/Sponsor to the Ethics and Health Research Authority within fifteen days.

### Protocol compliance

A member of the research team will be onsite or contactable after hours to advise and trouble-shoot and promote adherence to the protocol. Any accidental protocol deviations will be appropriately documented and reported to the Chief Investigator and Sponsor immediately. The Chief Investigator and sponsor will monitor and audit the conduct of this research.

## Research Ethics Committee (REC) and other regulatory review and reports

Before the start of the study, a favourable opinion was sought from a REC for the study protocol and other relevant documents (informed consent forms and patient information leaflets).

### Regulatory review and compliance

For any amendment to the study, the Chief Investigator or designee, in agreement with the sponsor will submit information to the appropriate body in order for them to issue approval for the amendment.

### Data protection and patient confidentiality

The General Data Protection Regulation and Data Protection Act 2018 will be adhered to. Data will be de-identified before being entered in a secure database. A unique study identifier number will be issued to each participant on enrolment into the study. That number will be used in all subsequent data collection forms. Patient paper data will be stored in a locked filing cabinet in a room that is only accessible to the research team and that is based at the Neonatal Intensive Care Unit facilities at King’s College Hospital. Patient de-identified electronic data will be stored on encrypted university computers or memory stick devices, both of which require a user identification and password verification.

### Archiving

At the end of the trial, all essential documentation will be archived securely by the CI for a minimum of 25 years from the declaration of the end of the trial. Essential documents include those which enable both the conduct of the trial and the quality of the data produced to be evaluated and show whether the site complied with all applicable regulatory requirements. All archived documents will continue to be available for inspection by appropriate authorities upon request.

### Indemnity

King’s College London indemnity applies for insurance/indemnity to meet the potential legal liability of the sponsor for harm to participants arising from the design and management of the research. NHS indemnity scheme applies for insurance/indemnity to meet the potential legal liability of the investigators arising from harm to participants in the conduct of the research.

### Access to the final study dataset

The individuals in the study will be notified of the outcome of the study as below. The investigators will have access to the final study dataset. Participants will be informed that anonymised data may be shared with other researchers for research purposes only.

### Dissemination policy

On completion of the study, the data will be analysed and a final study report will be prepared that could be accessed via the sponsor. The study will be presented at research meetings at the Neonatal Intensive Care Unit at King’s College Hospital as well as university meetings at King’s College London. Anonymised study data will be presented at conferences and published by the investigators in peer-reviewed journals. Participants will be notified of the outcome of the study via the provision of the publication and an accompanying newsletter.

### Authorship eligibility guidelines and any intended use of professional writers

The final study authors will include Dr Ourania Kaltsogianni, Dr Allan Jenkinson, Dr Theodore Dassios and Professor Anne Greenough. Professional writers will not be used.

### Intellectual property

All intellectual property rights and know-how in the protocol and in the results arising directly from the study shall belong to KCL.

## Discussion

This study will compare the effectiveness of closed-loop automated oxygen control to manual oxygen control in ventilated infants born at or above 34 weeks of gestation. To date, studies on CLAC have included very preterm or low birth weight infants [8–10, 16–21] and there are limited data on more mature neonates who are also vulnerable to the risks related to oxygen treatment. In a randomised crossover study, we demonstrated that the use of closed-loop automated oxygen control was associated with an increased percentage of time spent in the target oxygen saturation range and reduced time spent in hyperoxaemia, with fewer manual adjustments to the inspired oxygen concentration in ventilated infants born at or above 34 weeks of gestation [14]. In addition, CLAC was more effective in infants with more severe respiratory disease resulting in a larger increase in the time spent in oxygen saturation targets [11]. That study, however, was limited by its crossover design, the limited duration of the monitoring periods and the relatively low supplemental oxygen requirement of our infants. This randomised controlled trial will provide more robust evidence of the effect of CLAC on the duration of oxygen treatment that is related to respiratory morbidity [22] and the length of intensive care stay and therefore may demonstrate if CLAC could improve outcomes for late preterm and term born infants. This will be essential knowledge before the intervention is implemented into standard care.

Several algorithms have been developed for automated oxygen control and it is likely that differences in their design influence their performance. The ‘Oxygenie’ controller used in this study follows a proportional integral derivative (PID) algorithm. In preterm infants on respiratory support, the Oxygenie controller was more effective in keeping oxygen saturations within the target range and preventing hyperoxaemia and hypoxaemia when compared with a hybrid rule-based adaptive controller (CLiO_2_) [23, 24]. The use of an algorithm that most successfully avoids hypoxaemic and hyperoxaemic episodes will increase our ability to detect any differences in the primary outcomes of our study and may further reduce morbidity in our study population.

Participants are enrolled in our study within 24 h of initiation of mechanical ventilation. This limits the time frame to approach parents and obtain informed consent on participation but will provide more data points available for analysis as the duration of mechanical ventilation in infants born at or near term may be relatively short.

Previous studies investigating compliance with achievement of SpO_2_ targets showed that caregivers are more concerned to prevent hypoxaemia than hyperoxaemia with higher percentages of time spent above oxygen saturation targets [6, 25, 26]. Exposure to hyperoxia can lead to the generation of free radicals that induce membrane disruption and activate inflammatory pathways through lipid peroxidation, affecting multiple organ systems [27]. In addition, free radicals, as shown in animal studies, can activate several enzymes and vasodilators in the nitric oxide pathway promoting pulmonary vasoconstriction and resulting in persistent pulmonary hypertension of the newborn (PPHN) [28]. Newborn infants have reduced antioxidant capacity and they are particularly susceptible to the hyperoxic injury related to oxidative stress [29, 30]. Therefore, compliance with achievement of oxygen saturation targets and avoiding hyperoxaemia are important in near-term or term-born infants.

In conclusion, if this study demonstrates that CLAC reduces hyperoxia and the overall duration of oxygen treatment in more mature infants, that could help improve clinical outcomes and reduce morbidity in this population.

## Trial status

At the time of submission, this trial has been approved by the NHS Research Ethics Committee and the Health Research Authority (Protocol version 1.2, 10/11/2022). Recruitment of participants started on 01/12/2022 and will be completed by 30/11/2023.

## Supplementary Information

**Additional file 1. **SPIRIT checklist for trials.

## Data Availability

OK, TD, AJ and AG will have access to the final trial dataset. There are no contractual agreements that limit such access for all four investigators.

## Abbreviations

CI: Chief Investigator
CLAC: Closed-loop automated oxygen control
FiO2: Fraction of inspired oxygen concentration
HFOV: High-frequency oscillatory ventilation
HRA: Health Research Authority
KCH: King’s College Hospital
KCL: King’s College London
PID: Proportional-integral-derivative
PPHN: Persistent pulmonary hypertension of the newborn
ROP: Retinopathy of prematurity
REC: Research ethics committee
SAE: Serious adverse event
SpO_2_: Oxygen saturation levels
